# A63 FIRST REPORTED PEDIATRIC CASES OF SEWER WORKER’S DISEASE

**DOI:** 10.1093/jcag/gwae059.063

**Published:** 2025-02-10

**Authors:** M D Swatski, R M Issenman

**Affiliations:** Pediatric Gastroenterology, McMaster University Faculty of Health Sciences, Hamilton, ON, Canada; Pediatric Gastroenterology, McMaster University Faculty of Health Sciences, Hamilton, ON, Canada

## Abstract

**Background:**

Sewer worker’s disease, often recognized as a workplace hazard for sewer workers, has never been described in pediatric subjects.

**Aims:**

We report a case series of two siblings who developed chronic nasopharyngeal discharge, sore throat, diarrhea and irritability secondary to airborne supersaturation of *E. Coli* from an improperly vented household septic tank.

**Methods:**

Case Series- Retrospective Chart and Literature Review

**Results:**

The affected subjects were a six year old boy and his three year old sister. The boy presented with chronic diarrhea. The girl presented with six to eight watery stools per day and nocturnal stooling. Parents reported that both children also had a chronic purulent nasal discharge and complained of sore throats. Her parents also endorsed anorexia along with increased fussiness. Both siblings had been treated with metronidazole for *Giardia Lamblia* five years earlier. Repeat examination of stool for ova & parasites was negative. CBC and D-xylose were normal. Both children underwent upper GI endoscopy. Biopsy was normal in one and diffuse duodenal inflammation was noted in the second child. A heavy growth of E. *Coli* was cultured from the nares of both children.

The children both slept in a basement bedroom. Their father had installed a toilet in the basement 18 months prior to their presentation. The babysitter and the patient’s grandmother both developed similar symptoms after spending prolonged periods of time in the bedroom. Their symptoms resolved when they were away from the house. The mother recalled “a sewer” smell that had been present in the basement since her husband had installed a basement toilet. Professional inspection noted that the absence of a ”stack” to properly vent the toilet which was directly connected to the home septic tank. This left the basement air supersaturated with *E. Coli*. All the children’s symptoms resolved after the basement toilet was removed and the related drain sealed.

It is presumed that the growth of *E. Coli* as well as exposure to sewer gas was responsible for their ENT and GI symptoms. Lundholm, M. et al. describe how sewer worker’s symptoms including skin disorders and diarrhea were directly related to the concentration of airborne gram-negative bacilli.

As these children’s symptoms were directly related to exposure to an improperly vented sewage system and immediately resolved following corrective action, the most likely explanation was supersaturation. This should prompt Pediatricians to take a broader environmental history, particularly when multiple patients develop symptoms which are similar in character and time of onset.

**References:**

Lundholm, M., & Rylander, R. (1983). Work related symptoms among sewage workers. *Occupational and Environmental Medicine*, *40*(3), 325-329.

**Conclusions:**

When confronted with a cluster of common symptoms in uncommon places, physicians should consider the possibility of environmental diseases.

**Funding Agencies:**

**None**

